# Extraordinary Large Number of “Cysticercus Bovis” in a Calf Lung

**Published:** 1898-01

**Authors:** 


EXTRAORDINARY LARGE NUMRER OF "CYSTICERCUS BOVIS" IN A CALF LUNG.—As *cysticercus bovis* usually does not occur in large numbers, the present case is of especial interest. Everywhere on the lungs bright coffee-brown specks were visible, the centres of which were transparent and raised a little, and which showed a diameter of four millimetres. The microscopical examination showed that it was a case of *cysticercus bovis*. These parasites were found in the other organs, as follows: Heart, 7. In the liver a countless number beneath the serous coating of the front surface. On the tongue, 2. On the masseter, 3. On the muscles common to the head, neck, and arm, 1. On the lower shoulder-muscle, 3. Internal oblique abdominal muscle, 2. On the muscles of the thigh, a few. The diaphragm, spleen, kidneys, abdominal wall, and brain were free.—*Idem.*

